# Supplementary material for: Experimental observation of photonic nodal line degeneracies in metacrystals
Source: Nat Commun. 2018 Mar 5;9:950. doi: 10.1038/s41467-018-03407-5 (PMC5838093; doi:10.1038/s41467-018-03407-5)
Supplement: Supplementary file 1 — Supplementary Information [file 41467_2018_3407_MOESM1_ESM.pdf]

Supplementary information for Experimental observation of photonic nodal line degeneracies in metacrystals

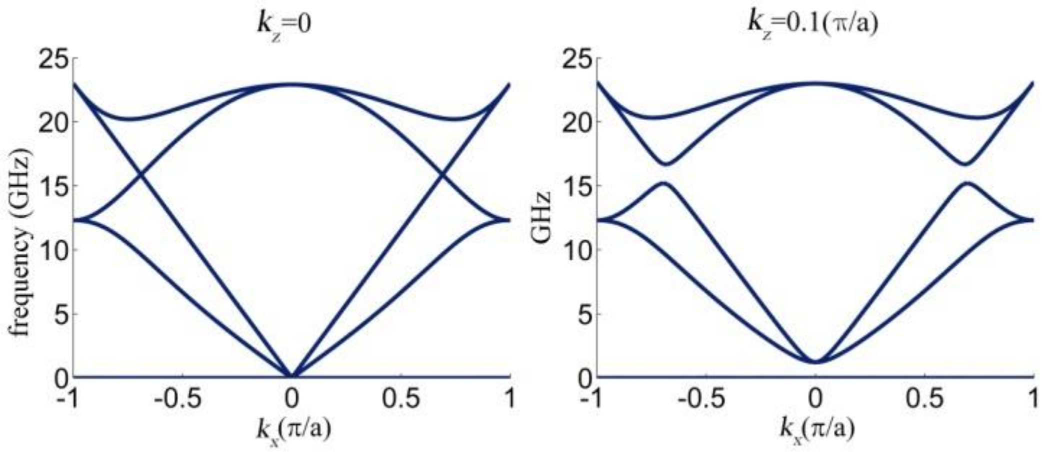

Supplementary Figure 1 Band structure calculated by the Hamiltonian formalism with zero and nonzero k_z

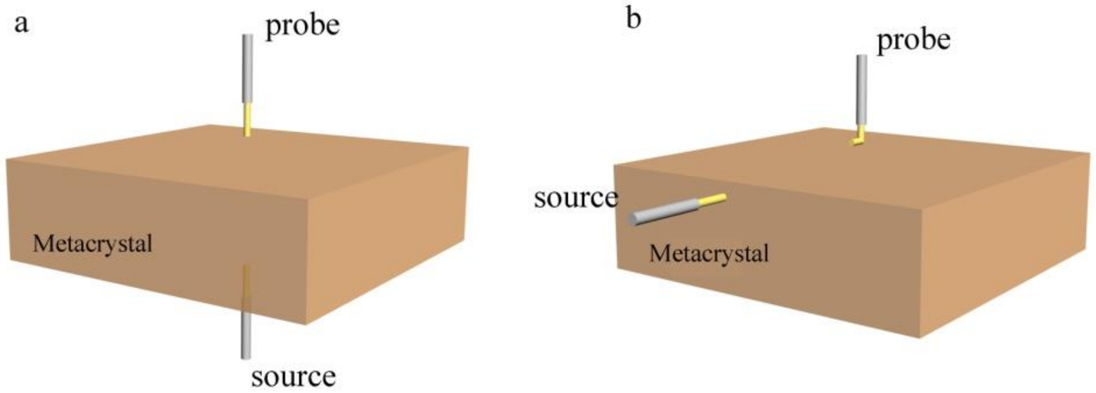

Supplementary Figure 2 Schematics of experiments for measuring (a) bulk state and (b) surface states of the metacrystal

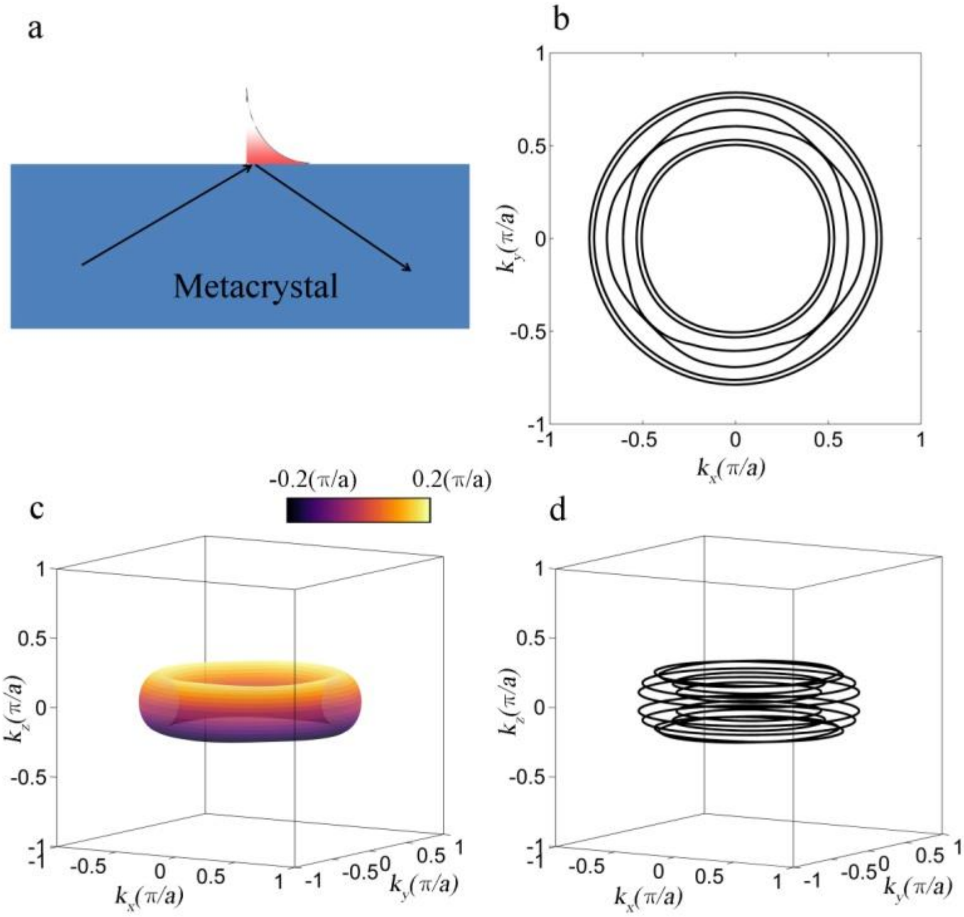

Supplementary Figure 3 Correspondence between the measured spectrum and band structure of the metacrystal. **a** The evanescent tails at reflection could be collected by a dipole antenna close to the surface. The colour in **c** denote the k_z value on the equi-frequency surface. Lines in **b** and **d** denote the states with discretized k_z value that could be measured in experiments.

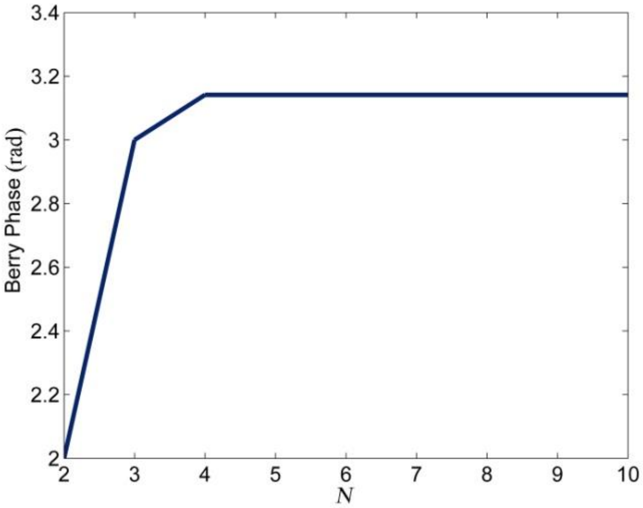

Supplementary Figure 4 Berry phase as function of total number of discretized points *N*.

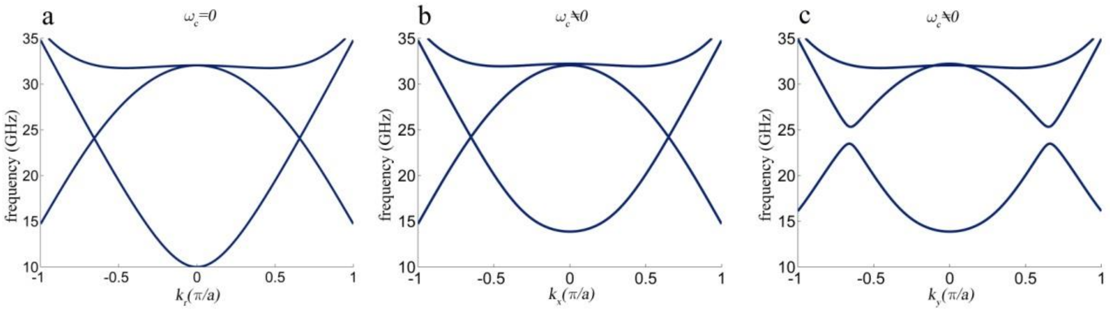

Supplementary Figure 5 Band structure with **a** zero and **b**-**c** non-zero ω_c calculated by the Hamiltonian formalism. **b** and **c** are calculated along x and y directions, signalling the emergence of Weyl points.

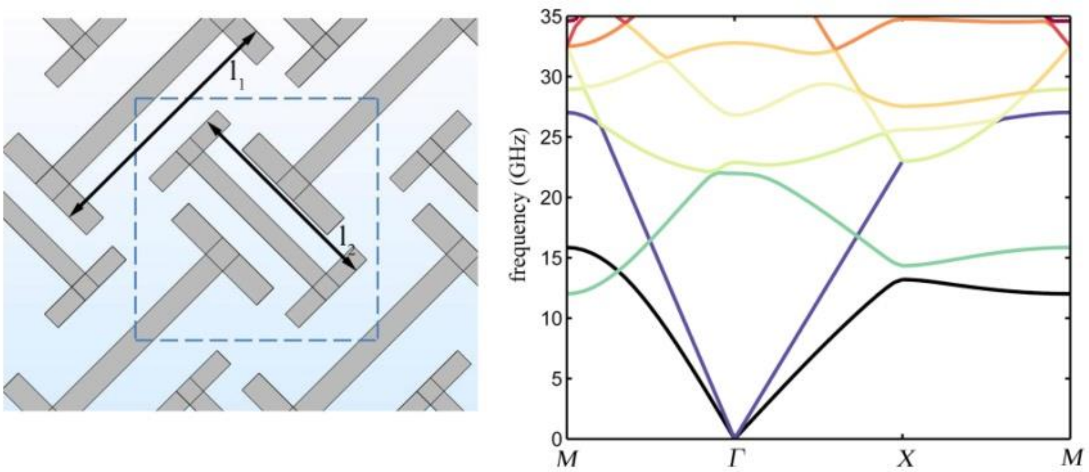

Supplementary Figure 6 Metamaterial design that breaks the glide mirror symmetry by setting $l_2=0.8*l_1$, while preserving the mirror symmetry $(x,y,z)\rightarrow(x,y,-z)$. The simulated band structure shows that the nodal line survives the perturbation.

Supplementary Table 1 Comparison between nodal lines and Weyl points

	Nodal lines	Weyl points
Dimension	1D	0D
Berry curvature	No	Yes
Density of states	$\lvert\omega-\omega_{NL}\rvert$	$\lvert\omega-\omega_{Weyl}\rvert^2$
Stability	stabilized by mirror symmetry	stable under any perturbation
Transition	Weyl points/ Normal Insulator	N/A

Some properties of nodal line and Weyl point emerged in the designed metacrystal are summarized in the Supplementary table 1. By introducing external magnetic field, the nodal lines could experience topological transitions and become WPs. If the mirror symmetry $(x,y,z)\rightarrow(x,y,-z)$ normal

to *z* axis is broken, the nodal lines will transform into a normal insulator with a trivial 3D photonic band gap. However, the Weyl points are topologically stable as source or drain as Berry curvature, and can't not be lifted by any symmetry lowering.

Supplementary Note 1. Hamiltonian formalism of the metacrystal

Following previous literatures on Hamiltonian formalism of dispersive photonic materials[1], the equation of motion in x-y plane where the resonators resides read:

$$\frac{d^2\mathbf{P}_{\parallel}}{dt^2}+\omega_0{}^2\mathbf{P}_{\parallel}=\omega_p{}^2\mathbf{E}_{\parallel} \tag{1}$$

Combining it with Maxwell equations renders the Hamiltonian:

$$\mathcal{H}\left|n\right\rangle=\omega\left|n\right\rangle \tag{2}$$

and

$$\mathcal{H}=\begin{bmatrix} 0 & i\varepsilon_s{}^{-1/2}\nabla\times & 0 & i\omega_p I_{3\times2} \\ i\varepsilon_s{}^{-1/2}\nabla\times & 0 & 0 & 0 \\ 0 & 0 & 0 & i\omega_0 \\ i\omega_p I_{2\times3} & 0 & -i\omega_0 & \end{bmatrix} \tag{3}$$

Note that $|n\rangle = [\mathbf{E}', \mathbf{H}', \mathbf{P}_{\parallel}{}', \mathbf{V}_{\parallel}{}'] = diag\left[\varepsilon_s, 1, \frac{\omega_0}{\omega_p}, \frac{1}{\omega_p}\right] * [\mathbf{E}, \mathbf{H}, \mathbf{P}_{\parallel}, \mathbf{V}_{\parallel}]$,. ε_s is the substrates permittivity 4.84. $I_{2\times3} = [I_{2\times2}, 0]$Unlike the parallel wire metacrystals, spatial dispersion of our non-symmorphic symmetry imposed metacrystal lacks a microscopic theory. We empirically let $\omega_p = 1 - \alpha(k_x^2 + k_y^2)$ to describe the spatial dispersion which is already satisfying enough describing the core physics underlying the nodal line in this type of metacrystal.

Band structure calculated by the Hamiltonian is given in Supplementary Figure 1. Here $\omega_p = 2\pi\times28\ GHz, \omega_0 = 0.44\omega_p$. *α*=0.005$\omega_p/c$, The longitudinal exhibit a negative dispersion as expected.

Supplementary Note 2. Experimental setup

For measuring bulk states in the metacrystal, a *z*-polarized electric dipole is placed at the centre of the bottom surface of the sample serving as the source, while another z-oriented dipole probe scans the top surface of the metacrystal to measures the E_z field component (Supplementary Figure 2a). For the surface states a y-polarized source dipole is placed close to the centre of one edge of the top surface of the metacrystal, while the field on the top surface is scanned by a y-polarized scanning probe dipole (Supplementary Figure 2(b)).

The source and probe antennas are connected to a Anritsu MS4647 vector network analyser. As the Probe scan the top surface of the metacrystal (step size is 4.5 mm for both x and y directions). Amplitude and phase information are collected by analysing the S-parameters, which could be further Fourier transformed into momentum space.

Correspondence between the measured spectrum and band structure of the metacrystal could be understood from the Supplementary Figure 3. As is shown in Supplementary Figure 3a, when

the bulk state in metacrystal is excited and propagate to the interface to air, it experience total internal reflection and is reflected back into the metacrystal. The evanescent tail in air during the total internal reflection process could be picked up by the near field antenna. Supplementary Figure 3c illustrate an example of equi-frequency contour. In the experiment, since the metacrystal has finite thickness in the z direction, the allowed k_z is discredited, as is demonstrated in Supplementary Figure 3d, where each loop represents a cut of the equi-frequency surface (EFS) in Supplementary Figure 3c of a definite k_z. Finally, since the fields are picked up at the upper boundary, only k_x k_y Fourier components can be extracted, but not k_z. The resultant measurement result should be the projection of the rings in Supplementary Figure 3d, as is shown in Supplementary Figure 3b. Thus the measurement results for different frequencies could provide information of the band structure, not that with definite k_z, but the projection with several different k_z.

Supplementary Note 3. Calculation of Berry phase of the nodal line by Wilson loop

With the Hamiltonian formalism well established, it's easy to calculate Berry phase of the nodal line by Wilson loop method. The Berry phase could be easily expressed by:

$$\theta = -ilog(\langle \varphi_1 | \varphi_2 \rangle \langle \varphi_2 | \varphi_3 \rangle .....\langle \varphi_N | \varphi_1 \rangle) \tag{4}$$

Here we set the loop threading the nodal line, and its radius equals $0.1\omega_p/c$. As is shown in Supplementary Figure 4, the Berry phase converges very fast to π as the number of discretized points on the loop N increases.

Supplementary Note 4. nodal line metacrystal with gyroelectric material

Upon applying the gyroelectric material (magnetized plasma) into the metacrystal, and assume resonance of the cut wires are not changed. Hamiltonian formalism of the metacrystal changes to:

$$\mathcal{H} = \begin{bmatrix} 0 & i\hat{Q}\nabla\times & 0 & i\omega_p I_{3\times2} & -i\omega_{p2} I_{3\times2} \\ i\nabla\times\hat{Q} & 0 & 0 & 0 & 0 \\ 0 & 0 & 0 & i\omega_0 & 0 \\ -i\omega_p I_{2\times3} & 0 & i\omega_0 & 0 & 0 \\ -i\omega_{p2} I_{3\times3} & 0 & 0 & 0 & \omega_c\hat{\sigma}_y \end{bmatrix} \tag{5}$$

and $\widehat{\sigma_y} = [0,0;0,\sigma_y]$. ω_{p2}=$\omega_c = 2\pi * 10GHz$ are plasma frequency and cyclotron frequency of the magnetized plasma, respectively. Basis of this new Hamiltonian is $[\mathbf{E}, \mathbf{H}, \mathbf{P}_{||}, \mathbf{V}_{||}, \mathbf{S}]$ where **S** account for the velocity field in magnetized plasma. When external bias magnetic field is not applied, band structure is given in Supplementary Figure 4. The nodal is still at present, which agrees reasonably well with Figure 5a in the main text.

Once the magnetic field is applied, the nodal line can also degenerate into Weyl points (WP) in the Hamiltonian model, as is given in Supplementary Figure 5. The mismatch between effective Hamiltonian and the real structure, especially near resonance frequency of the magnetized plasma comes from the fact that the change of resonance of the cut-wires are not considered in the Hamiltonian model for simplicity. However, behaviour around the nodal line agrees reasonably well

between them.

Supplementary Note 5. Effective Hamiltonian model of the Weyl point and nodal line in metacrystal

To construct effective Hamiltonian of the WP, we firstly express eigen states at the WP as

$$\begin{aligned} |I\rangle &= \left[0,e_y,e_z,0,h_y,h_z,0,p_y,0,v_y,0,s_y,s_z\right]^T \\ |J\rangle &= \left[e_x,0,0,0,0,0,p_x,0,v_x,0,s_x,0,0\right]^T \end{aligned} \tag{6}$$

The first order expansion around the Weyl point is thus:

$$\mathcal{H}_1 = [\langle I(\boldsymbol{k}_0)|\frac{\partial\mathcal{H}}{\partial\boldsymbol{k}}d\boldsymbol{k}|J(\boldsymbol{k}_0)\rangle + \omega(\boldsymbol{k}_0)\delta_{IJ}] \tag{7}$$

substituting supplementary equation (6) into supplementary equation (7), we have the matrix form effective Hamiltonian:

$$\mathcal{H}_1 = \begin{bmatrix} 2Re(e_y h_z^* - e_z h_y^*)k_x + 4\alpha^2 mRe(ie_y v_y^*)k_x & -e_x h_y^* k_z + e_x h_z^* k_y \\ -e_x^* h_y k_z + e_x^* h_z k_y & -4\alpha^2 mRe(ie_x v_x^*)k_x \end{bmatrix} \tag{8}$$

where $k_{x,y,z}$ is small deviation of momentum from the WP, and m is distance to the WP from Brillouin zone center. Letting e_x, h_y real and h_z imaginary, we have:

$$\begin{aligned} &\mathcal{H}_1 = v_1\sigma_x k_z + (v_2\sigma_z + v_3 I)k_x + v_4\sigma_y k_y \\ &v_1 = -Re(e_x h_y) \\ &v_2 = Re(e_y h_z^* - e_z h_y^*) + 2\alpha^2 mRe(ie_y v_y^*) + 2\alpha^2 mRe(ie_x v_x^*) \\ &v_3 = Re(e_y h_z^* - e_z h_y^*) + 2\alpha^2 mRe(ie_y v_y^*) - 2\alpha^2 mRe(ie_x v_x^*) \\ &v_4 = Re(e_x)Im(h_z) \end{aligned} \tag{9}$$

which is a Weyl Hamiltonian.

Subtract the static magnetic field from the metacrystal, the eigen states are changed to:

$$\begin{aligned} |I\rangle &= \left[0,0,e_z,0,h_y,0,0,0,0,0,0,s_y,0\right]^T \\ |J\rangle &= \left[e_x,0,0,0,0,0,p_x,0,v_x,0,s_x,0,0\right]^T \end{aligned} \tag{10}$$

and the supplementary equation (9) reduces to:

$$\begin{aligned} &\mathcal{H}_1 = v_1\sigma_x k_z + (v_2\sigma_z + v_3 I)k_r \\ &v_1 = -Re(e_x h_y) \\ &v_2 = Re(-e_z h_y^*) + 2\alpha^2 mRe(ie_x v_x^*) \\ &v_3 = Re(-e_z h_y^*) - 2\alpha^2 mRe(ie_x v_x^*) \end{aligned} \tag{11}$$

which is the equation (1) in the main text.

Furthermore, since velocity field S is not at present in the PCB board configuration (Fig. 1a in main text), it is possible to derive the eigen states on nodal line (NL), which are:

$$
\begin{aligned}
&\left|I\right\rangle = \frac{\sqrt{2}}{2}\left[0,0,-1,0,1,0,0,0,0,0\right]^T \\
&\left|J\right\rangle = \eta^{-1/2}\left[\omega_p,0,0,0,0,0,\omega_0,0,i\omega_{NL},0\right]^T \\
&\eta = \omega_p^{\ 2} + \omega_0^{\ 2} + \omega_{NL}^{\ 2}
\end{aligned}
\tag{12}
$$

and supplementary equation (11) becomes:

$$
\mathcal{H} = \sqrt{\frac{\omega_p^{\ 2}}{2\varepsilon_s\eta}}\sigma_x k_z + \{\sqrt{\frac{1}{\varepsilon_s}}(\frac{1}{2} + 2\alpha^2 m\frac{\omega_p\omega_{NL}}{\eta})\sigma_z + \sqrt{\frac{1}{\varepsilon_s}}(\frac{1}{2} - 2\alpha^2 m\frac{\omega_p\omega_{NL}}{\eta})I\}k_r
\tag{13}
$$

When k_z=0, the Hamiltonian is diagonal:

$$
H = \begin{bmatrix} \sqrt{\frac{1}{\varepsilon_s}}k_r & 0 \\ 0 & -4\alpha^2 m\frac{\omega_p\omega_{NL}}{\sqrt{\varepsilon_s}\eta^2}k_r \end{bmatrix}
\tag{14}
$$

and the diagonal entries represents the positive and negative linear dispersion relations of the TE mode and longitudinal mode.

Supplementary Note 6. Nodal line's stability under mirror symmetry preserving perturbations

The NL is formed by the degeneracy between the longitudinal mode and the transverse electric mode. The existence of the NL is protected by the mirror symmetry instead of the glide mirror symmetry. Here the glide symmetry is utilized to introduce degeneracy between the LP mode and the TM mode at the Brillouin zone edge, ensuring a negative dispersion for the LP mode and a clean nodal line in the momentum space free of other bulk modes at the same frequency. Breaking of the glide symmetry does not lift the degeneracy, which is illustrated by the band structure of the metamaterial with broken glide symmetry by shrinking the size of one of the cut wire resonator ($l_2=0.8*l_1$) in Supplementary Figure 6.

Note that although the doubly degeneracy at Brillouin zone boundary enabled by the glide reflection symmetry is lifted, the NL degeneracy persists. However, in this configuration the band structure close to the nodal line frequency is not clean as that with glide mirror symmetry (Fig. 1d of the main text). Nevertheless, any deformations apart from the mirror symmetry breaking will not lift the NL degeneracy.

Supplementary References

[1] Raman, A. & Fan, S. Photonic Band Structure of Dispersive Metacrystals Formulated as a Hermitian Eigenvalue Problem. Phys. Rev. Lett. **087401** (2010).
